# Defining the imaging diagnostic criteria for adult chronic non-bacterial osteitis

**DOI:** 10.1093/jbmrpl/ziae024

**Published:** 2024-03-08

**Authors:** Ashna I E Ramautar, Ana Navas, Elizabeth M Winter, Herman M Kroon, Frits Smit, Dennis Vriens, Neveen A T Hamdy, Natasha M Appelman-Dijkstra

**Affiliations:** Centre for Bone Quality, Division of Endocrinology, Department of Internal Medicine, Leiden University Medical Center, Leiden, The Netherlands; Section of Musculoskeletal Radiology, Department of Radiology, Leiden University Medical Center, Leiden, The Netherlands; Centre for Bone Quality, Division of Endocrinology, Department of Internal Medicine, Leiden University Medical Center, Leiden, The Netherlands; Section of Musculoskeletal Radiology, Department of Radiology, Leiden University Medical Center, Leiden, The Netherlands; Section of Nuclear Medicine, Department of Radiology, Leiden University Medical Center, Leiden, The Netherlands; Department of Nuclear Medicine, Alrijne Hospital, Leiderdorp, The Netherlands; Section of Nuclear Medicine, Department of Radiology, Leiden University Medical Center, Leiden, The Netherlands; Centre for Bone Quality, Division of Endocrinology, Department of Internal Medicine, Leiden University Medical Center, Leiden, The Netherlands; Centre for Bone Quality, Division of Endocrinology, Department of Internal Medicine, Leiden University Medical Center, Leiden, The Netherlands

**Keywords:** rare bone disease, (auto) inflammation, sclerosis, hyperostosis, calcified ligaments, computerized tomography, skeletal scintigraphy, CNO, osteitis

## Abstract

Osteitis of the sternocostoclavicular (SCC) region, referred to as sternocostoclavicular hyperostosis (SCCH), is the clinical expression of chronic non-bacterial osteitis (CNO) in adults with this rare chronic auto-inflammatory disorder of the axial skeleton. The diagnosis is based on distinctive computerized tomography (CT) features of sclerosis and hyperostosis of the SCC region, and local increases in osteoid formation visualized by high radiopharmacon uptake on skeletal scintigraphy but clear radiologic diagnostic criteria are lacking. In a cross-sectional study, CT scans and whole-body skeletal scintigraphy images obtained in 169 patients seen at the Center for Bone Quality of the Leiden University Medical Center between 2008 and 2018 with a suspected diagnosis of CNO of the SCC region were re-evaluated by 2 skeletal radiologists and 2 nuclear physicians. The diagnosis was confirmed in 118 (70%) predominantly female patients (*n* = 103, 89.2%); median age at first symptoms 45 years (range 20-73). The diagnosis was excluded in the remaining 51 “non-CNO” patients. Increased radiopharmacon uptake at the SCC region was observed in 82% CNO patients, with the manubrium sterni having the highest predictive ability to discriminate on both imaging modalities. The prevalence of sclerosis of the clavicles, manubrium and first ribs was significantly higher in CNO patients (*P* < 0.001). Hyperostosis was not observed in non-CNO patients. 46 CNO versus only 2 non-CNO patients had costoclavicular ligament calcification. Our findings identify CT scan features of sclerosis and hyperostosis of manubrium sterni, medial end of clavicles and first ribs, and calcification of costoclavicular ligaments, associated with increased tracer uptake on skeletal scintigraphy at the SCC region, specifically manubrium sterni, as well-defined imaging diagnostic criteria for adult CNO. Pitfalls encountered in the diagnosis of CNO are highlighted. These defined imaging diagnostic criteria for adult CNO should facilitate the diagnosis of this rare auto-inflammatory bone disease across the spectrum of its early to late stages.

## Introduction

Osteitis of the sternocostoclavicular (SCC) region, referred in the literature as sternocostoclavicular hyperostosis (SCCH), is the most common clinical expression of chronic non-bacterial osteitis (CNO) of the anterior chest wall (ACW) in adults with this rare chronic auto-inflammatory disorder of the axial skeleton, the precise pathophysiology of which remains elusive. The natural history of CNO is characterized by recurrent episodes of exacerbation and remission in its early stages, with progressive shortening of the periods of remission, eventually leading to a chronic state with potentially debilitating symptoms. Clinical features include local inflammatory changes such as pain, redness, swelling, warmth, and impairment of shoulder girdle function.^1-3^ The diagnosis is principally based on distinctive computed tomography (CT) scan features of sclerosis, and hyperostosis, characteristically in bones of the SCC region, associated in later stages of the disease with calcification of ligaments and eventually possible ankylosis of adjacent joints. Increased bone resorption may be observed in the form of cortical bone erosions and/or intraosseous lucencies in earlier stages of the disease. Other areas of the axial skeleton such as mandible, spine, and/or bones of the sacroiliac region may also be affected^1^^,^^4-6^ However, clear imaging diagnostic criteria are lacking and are as yet to be established. The term “bull head sign” describing the characteristic increased bilateral uptake of radiopharmacon at the medial end of both clavicles and manubrium sterni observed in some CNO patients was first coined in the late 1990s to the skeletal scintigraphy pattern, and is still considered by some authors to be the imaging hallmark of osteitis of the SCC region.^7^^,^^8^ However, in a recent publication from our group over a relatively large cohort of patients with adult CNO, we observed this sign to be only present in a minority of patients,^1^ supporting its low prevalence as also reported by Fu et al.^9^ although others reported its presence in up to 40% of cases.^10^

This discrepancy in prevalence is likely to be due to the different criteria used to define this distinctive sign, including incomplete bullhead sign, rather than the originally described complete uptake pattern. In this study, we have used the definition of the bull head pattern as originally described in the literature, which has also been consistently used by our radiologists over the years.

Whole body skeletal scintigraphy also provides additional information about the distribution of the disorder in other areas of the axial skeleton such as spine, mandible, and sacroiliac region, which may be affected in 30-60% of cases.^1^^,^^5^^,^^6^^,^^10^

The current state of radiological evaluation consists of two approaches for the classification of CNO, developed for the assessment of extent and severity of the disorder. The initial staging of adult CNO was proposed by Sonozaki et al. in the late 1970s and was based on plain conventional radiography demonstrating mild ossification of the SCC region (stage 1).^11^ This staging was subsequently modified in the early 1990s by adding the involvement of multiple ribs, most reflected by the spread of ossification towards the first ribs (referred to as stage 2), and ossification of other affected axial localizations. This modification proposed that involvement of any rib beyond the seventh should decrease suspicion for CNO of the SCC region, as this would be more likely to be due to generalized calcification of the cartilage of ribs.^12^^,^^13^ The Sonozaki staging was also adapted for the more sensitive imaging obtained by CT scan, which enabled the detection of abnormalities in de SCC region not obvious on plain radiographs. A further classification of radiological findings proposed by Chigira and Shimizu in the late 1980s added a stage 0 to the Sonazaki staging, as defined by the absence of clearly demonstrable ossification at various sites of the SCC region.^14^ Although not fully concordant, both classifications take into consideration the progression of calcification/ossification of the SCC region including osseous malformations and ligamentous and cartilaginous abnormalities, such as ossification of the costoclavicular ligaments, and of the cartilaginous surface of first ribs and surrounding tissue, with loss of definition of inferior and superior margins of the clavicles and first ribs.^11^^,^^12^^,^^14^^,^^15^

Although these proposed radiographic staging systems have been used as guidance for clinicians and radiologists for the diagnosis of CNO of the SCC region, albeit not universally, this diagnosis remains challenging in the absence of definitive radiological diagnostic criteria for both CT and skeletal scintigraphy. Several patients with this disorder remain thus to date underdiagnosed and untreated, particularly in the early stages of the disease, with prolonged delays in diagnosis often associated with the development of chronic changes, persistent pain and limitation of function, significantly impacting on several aspects of quality of life.^16^ This further stresses the need for a clear set of diagnostic imaging criteria for the disease.

The main objective of this cross-sectional study was to identify and define the characteristic radiologic and scintigraphic features of adult CNO in patients with an established diagnosis for the disorder. This should facilitate early diagnosis of CNO, thereby allowing early initiation of treatment, and exclusion of the diagnosis in the absence of these features, as tested in a control group of patients in whom the disorder was excluded. The second objective of the study was to establish the need to also evaluate non-structural features of the disease such as disease activity, which in our study was achieved by using whole body ^99m^Technetium-diphosphonate (HDP) skeletal scintigraphy. Areas of increased radioisotope uptake at skeletal sites outside the SCC region were identified as active lesions, which were sometimes clinically silent. Possible associated structural changes in these latter sites were subsequently confirmed by CT scan.

Throughout this article, we refer to CNO of primarily affecting the SCC region as just adult “CNO” rather than using the previously used nomenclatures of “isolated SCCH” or “CNO/SCCH,” based on recommendations from a recent international consensus meeting on adult CNO, at which it was recommended that these current nomenclatures should be abandoned for that of “Adult CNO” (manuscript in preparation).

## Material and methods

### Study design

This was a single center cross-sectional study re-evaluating digital radiological and scintigraphic images obtained between 2008 and 2018 in a cohort of 184 consecutive adult patients with a suspected diagnosis of CNO at referral to the Center for Bone Quality of the Leiden University Medical Center, a Dutch national expertise center for Metabolic and Rare Bone Diseases.

Inclusion criteria were availability of complete sets of clinical, biochemical, and imaging data including whole-body skeletal scintigraphy, a diagnostic CT scan of affected region(s) or skeletal scintigraphy combined with single photon emission computed tomography (SPECT-CT) of the affected region(s).

A diagnosis of CNO had been established and subsequently confirmed by an experienced clinician in 70% of subjects included in the study (*n* = 118) on the basis of distinctive clinical features as previously described in our large CNO cohort study (local inflammatory changes in the SCC region such as pain, redness, swelling, warmth, and impairment of shoulder girdle function), and a spectrum of radiological features in the same region including, according to the stage of the disease, sclerosis, hyperostosis, bone erosions, intraosseous lucencies, calcification of ligaments, and ankylosis of adjacent joints. The diagnosis was supported by the natural history of the disorder, normal biochemistry panel (renal and liver function, serum electrolytes, calcium, and phosphate) normal inflammatory markers (CRP and ESR). Bone turnover markers (ALP and P1NP) were measured to exclude possible other metabolic bone diseases.^1^ The presence of the HLA B27 antigen was tested in case of clinical suspicion of a spondyloarthropathy. A diagnosis of CNO was excluded in the remaining 30% of patients not demonstrating these features (non-CNO group, *n* = 51), in whom another pathology such as osteoarthritis (OA), inflammatory arthritis, spondyloarthropathy, congenital disorders, recurrent subluxation of the clavicle or fracture of the clavicle was diagnosed (Figure S1).

### Patients

All 169 patients included in the study had available and complete sets of clinical, biochemical, and evaluable radiological and scintigraphic digital images including a whole-body skeletal scintigraphy using ^99m^Tc-labelled HDP, and a diagnostic CT of the SCC region and other affected regions of the axial skeletal (carried out at different but not widely spaced times) demonstrating increased radioactive isotope uptake. From 2014 onwards, CT of the ACW was routinely performed combined with scintigraphy (SPECT-CT). Demographic and clinical data retrieved from the patients’ electronic medical records included data on sex, age at first symptom(s) and at diagnosis, delay in diagnosis (the interval between first symptoms and diagnosis), extra-skeletal manifestations such as palmoplantar pustulosis and/or presence of autoimmune diseases, and biochemical data. Data on clinical manifestations (specifically pain, local inflammatory changes and limitation of shoulder function) were also retrieved from the medical records.

Four experienced imaging specialists: 2 skeletal radiologists, and 2 nuclear physicians, blinded for the final diagnosis and original imaging reports, independently re-evaluated original diagnostic imaging scans, using a predefined set of criteria built in a scoring list developed by the study team (Appendix A), based on data from previously published literature and on the collective personal experience and observations of the study investigators and authors.^1^^,^^6^^,^^9^^,^^11^^,^^13^^,^^14^^,^^17^^,^^18^ The investigators did not have access to patients’ files for follow-up images or reports. Radiologists were blinded for scintigraphic findings, and nuclear physicians for radiological findings. All scintigraphic and CT imaging scans were evaluated independently and separately by a single radiologist or a single nuclear physician, with one of the other researchers as a note-taker. Data were compared between CNO and non-CNO patients.

### Inter-observer variability

A randomly chosen subset of 10% of all scans was independently evaluated prior to the start of the study by all 4 observers to assess inter-observer variability, which was separately calculated for radiologists and nuclear medicine physicians.

Differences in the evaluation of the 10% of all scans were also resolved during a consensus meeting, after which a second randomly chosen different subset of 10% of all scans was again independently evaluated by all 4 observers and a second consensus meeting was held prior to the start of the study.

### Re-evaluation of imaging scans

The ^99m^Tc-HDP whole body skeletal scintigraphy was acquired in all patients using standardized procedures for our center, and as per referring center protocols when performed elsewhere. Image acquisition adhered to the Dutch and European Association for Nuclear Medicine practice guidelines for skeletal scintigraphy. Parameters evaluated were increased radiopharmacon uptake in the anatomical distribution of lesions, and quantification of degree of skeletal involvement, in excess to those physiologically expected such as in growth plates in young adults.

**Figure 1:**
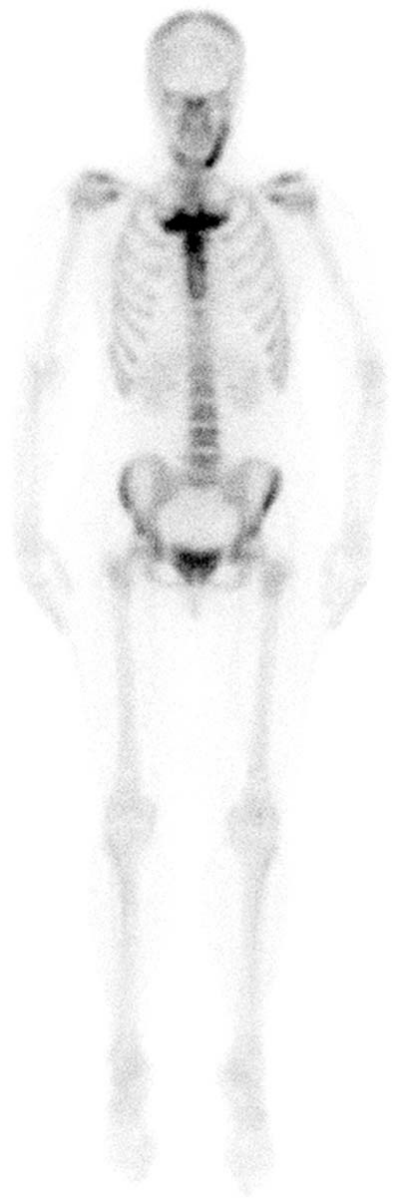
Distinctive imaging features of CNO of the SCC region and mandible. Skeletal scintigraphy using 99mTc-HDP demonstrating increased radiopharmacon uptake of the SCC region at the level of the sternal manubrium and proximal corpus sterni, medial end of first ribs and left mandible.

A bull head pattern was reported in the presence of the previously described characteristic bilateral increased uptake of the radiopharmacon at the medial end of both clavicles and manubrium sterni, with the manubrium sterni representing the bull’s upper skull and the medial end of clavicles and first ribs bilaterally corresponding to the bull’s horns (Figure 1).

**Figure 2:**
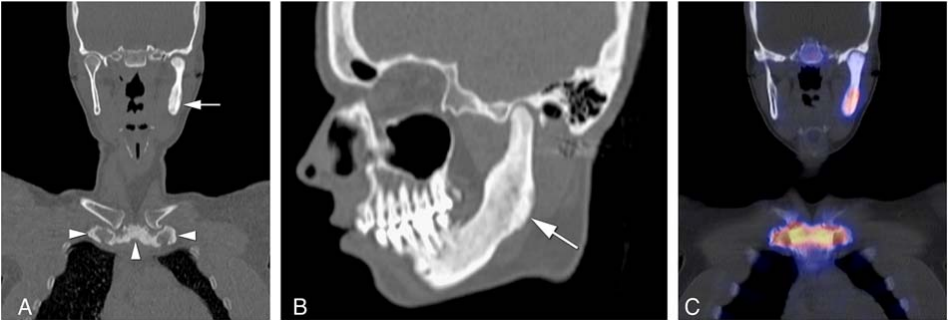
(A) Radiologic features of sclerosis and hyperostosis of the sternal manubrium and end of first ribs (arrowheads) and in the left mandible (arrow), on coronal reconstruction of computed tomography (CT) of the same patient as in Figure 2. Both clavicles are normal. (B) Sagittal reconstruction of CT of the same patient shows sclerosis and mild hyperostosis of the left mandible (arrow). (C) Increased uptake on SPECT-CT corresponding with increased osteoblastic activity (bone formation) is seen in the left mandible, sternal manubrium and medial ends of first ribs.

**Figure 3:**
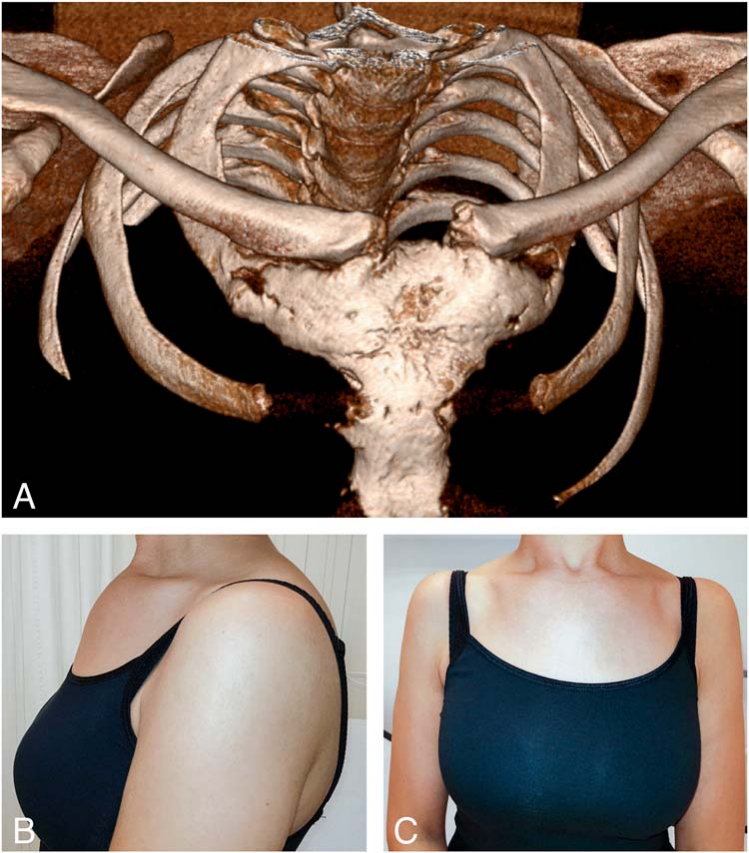
(A) 3D image reconstruction of a CT scan of the sterno-costo-clavicular region in a different patient with CNO showing bilateral hyperostosis of the manubrium sterni at the level of the first rib forming a large bony costo-manubrial bridge. Hyperostosis of the proximal right clavicula and ankylosis of the manubrio­ sternal joint can also be seen. (B and C) Frontal and lateral clinical images obtained at diagnosis in the same patient showing bone enlargement in the SCC region of the anterior chest wall.

Computerized tomography scans of the ACW were performed in all patients, with other areas of the axial skeleton showing increased radiopharmacon uptake on ^99m^Tc-HDP scintigraphy such as mandible, spine or sacroiliac (SI) region additionally scanned as illustrated in the Figure 1 patient (Figure 2A-C). The 3D CT reconstruction of Figure 2A and C, and corresponding clinical images are shown in Figure 3A-C).

Both radiologists were asked in separate sessions and blinded from each other, whether the changes they observed were compatible with a diagnosis of CNO. In case of a negative response, they were requested to provide an alternative diagnosis to CNO.

**Figure 4:**
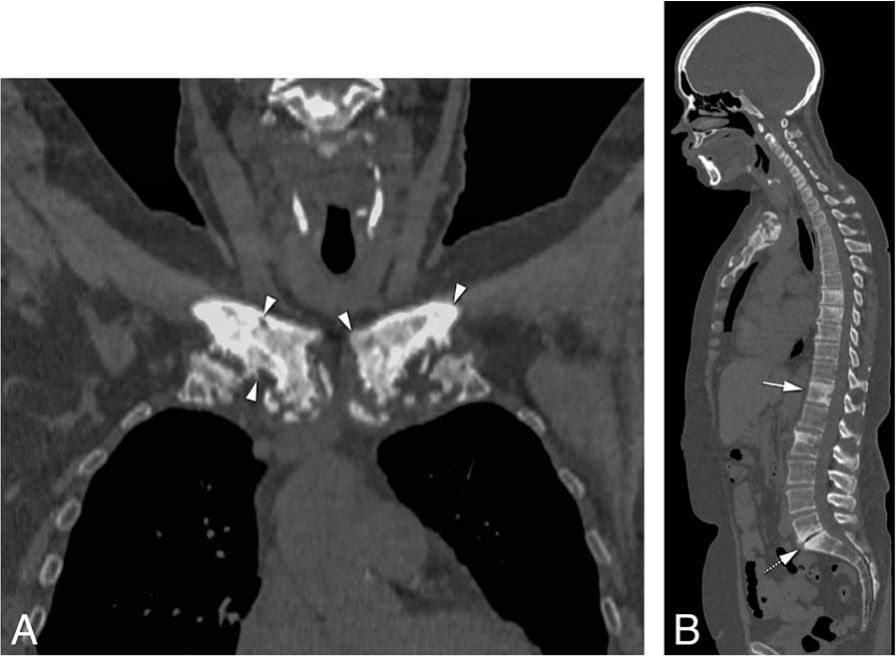
(A) Features of sclerosis, hyperostosis, and bone erosions of the medial end of clavicles and first ribs on coronal reconstruction of CT scan (arrowheads). (B) Sagittal image of CT scan showing characteristic patchy areas of sclerosis and mild hyperostosis of thoracic (T8, T9, TIO, T12) and lumbar (L2) vertebrae consistent with osteitis (arrow at T12 level). These features are different from reactive “kissing” vertebral plate sclerosis (Modic 3 changes) indicating degenerative changes associated with narrowing of the intervertebral space, intervertebral disk vacuum phenomenon, and absence of hyperostosis observed at L5/S1 (dashed arrow). The lesions were active on skeletal scintigraphy (not shown).

**Figure 5:**
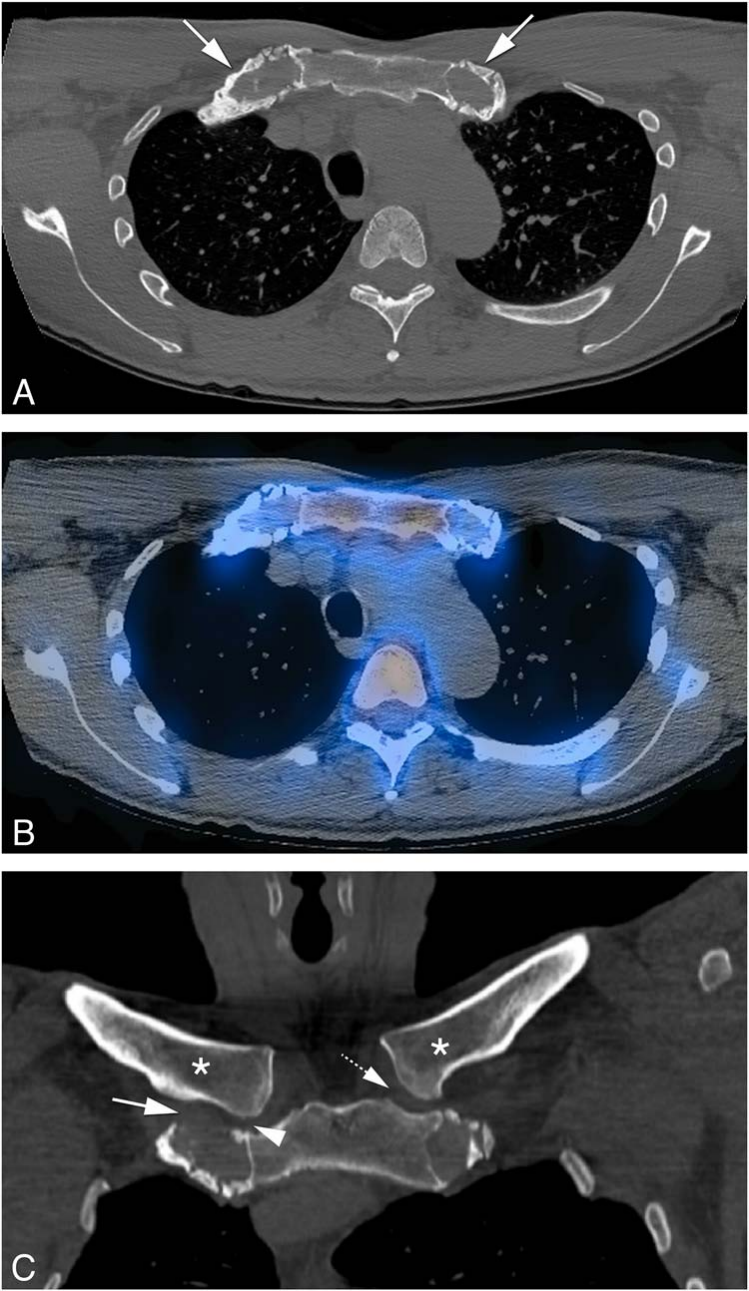
(A) Bilateral hypertrophic ossification of the cartilage and costosternal junctions of the first ribs (arrows). (B) Corresponding SPECT-CT imaging showing no increased uptake in these areas on CT scan, indicating no increase in bone remodeling. (C) Coronal CT imaging showing no abnormalities in the clavicles bilaterally, specifically no sclerosis or hyperostosis (asterisks), no costoclavicular ligament calcification (arrow), and no narrowing of the sterno-clavicular joint space (arrowhead right and dashed arrow left).

**Figure 6:**
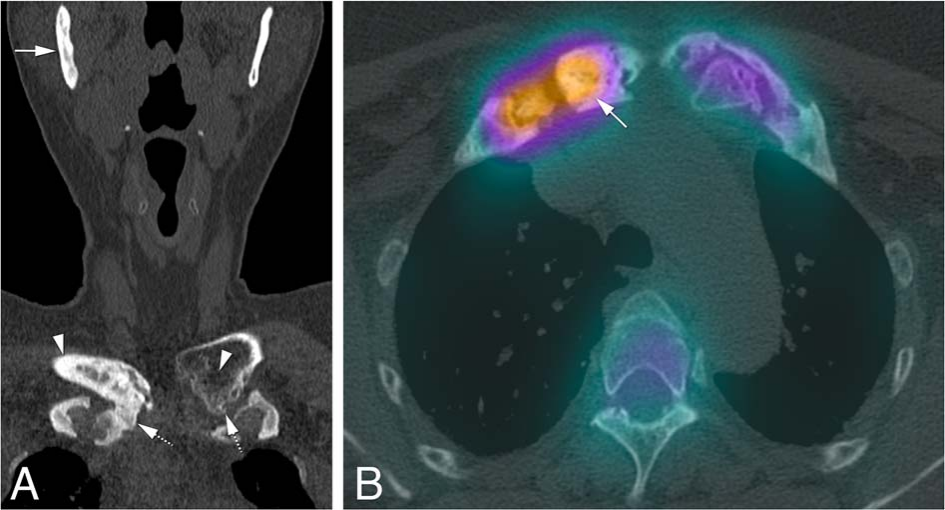
(A) Subtle CNO features at the level of the medial end of the left clavicle in the form of endomedullar bony lucencies (arrowhead), and subtle calcification of the costoclavicular ligament (arrow), while a more advanced stage of the disease can be seen in the contralateral medial end of the right clavicle in the form of sclerosis, hyperostosis and bone erosions (arrowhead), as well as partial ankylosis of the right costoclavicular joint (arrow). Sclerosis and hyperostosis is also seen in the right mandible (arrow). (B) Increased radiopharmacon uptake on SPECT-CT of the right clavicle in the presence of hyperostosis reflecting increased bone formation as also seen in (A), in contrast with the absence of increased radiopharmacon uptake on the contralateral side, which demonstrated only mild abnormalities at the left side.

In this study, CT scans were analyzed according to the presence of the following, conventionally defined CT features observed in the affected regions of the axial skeleton:

- Sclerosis: localized or generalized increase in bone density on CT scan of affected area(s) due to abnormal thickening of trabecular (spongy, cancellous) bone. In the context of CNO, sclerosis is the radiological expression of an (auto)-inflammatory osteitis induced by increased bone turnover in affected areas of the skeleton with increased bone formation in excess of bone resorption depending on the stage of the disease. Sclerosis may be present at any affected site of the axial skeleton and not just limited to subchondral bone. A common CT radiological interpretative pitfall is to differentiate between the subchondral reactive sclerosis limited to adjacent joints observed in the sternoclavicular region or around the (SI) region due to OA, usually associated with other radiological manifestations such as osteophytes, subchondral cyst formation and asymmetrical narrowing of the affected joint space, and the more extensive sclerosis observed in CNO.
  In case of spondylarthrosis, spinal reactive “kissing” vertebral plate sclerosis can be observed due to disk degeneration, usually associated with narrowing of intervertebral spaces (Modic type 3 changes) and spondylophytes (Figure 4B).
- Hyperostosis: abnormal widening of cortical (compact) bone due to periosteal reaction with cortical thickening causing an increase in the volume and size of the affected bone (bone hypertrophy). This frequently occurs in affected bones of the SCC region (especially in the clavicle) and mandible and less frequently in the spine (Figures 1, 2A and B, and 4B).
  In this study, we specifically define the term “hypertrophic ossification,” as a pattern of calcification/ossification often described as part of the aging process, strictly affecting the costochondral or costosternal junctions, mostly of the first ribs,^19^^,^^20^ without associated sclerosis of the adjacent bones, characteristically sparing bones of the SCC region of the ACW, eventually leading to ankylosis of costosternal and/or costochondral joints. (Figure 5A-C). This radiological pattern should be distinguished from the pattern of involvement associated with CNO and represents a pitfall for this diagnosis. Follow-up may be necessary in case of development of symptoms, or in doubtful cases.
- Bone erosions are seen on CT scan as small ill-defined osteolytic lesions without sclerotic margins, located in the subchondral bone plate due to cortical bone defects with alterations of the adjacent trabecular bone, or as intraosseous trabecular bone lucencies that develop due to excessive local bone resorption, not yet matched by adequate bone formation. These osteolytic lesions are likely to represent an inflammation-driven early phase radiological feature preceding the development of sclerosis and hyperostosis in its more advanced stages (Figure 6A and B). However, cortical bone erosions are not distinctive features of adult CNO and they may represent a pitfall for its diagnosis as they can be frequently observed in other inflammatory arthropathies such as erosive OA, SpA, rheumatoid arthritis (RA), or in the context of crystal deposition disease (CPPD arthropathy) such as uric acid deposition in gout. A further interpretative pitfalls on CT scan are subchondral bone cysts*.* These are focal bony lucencies in the subchondral bone plate most frequently due to OA, which unlike bone erosions have sclerotic margins. Bone cysts are also usually most prominent in the inferior aspect of the medial clavicle and are normally observed associated with other radiological manifestations of OA such as osteophyte formation and (asymmetrical) narrowing of the joint space.
- Calcification/ossification of tendons/capsules/joints: In pathological “calcification,” calcium salts are deposited in normal tissue (metastatic calcification) or damaged tissue (dystrophic calcification), whereas the term “ossification” implies bone formation (calcification in a collagen matrix).^21^ The two processes are in many cases radiographically indistinguishable as pseudotrabeculation may occur with calcification.^22^ In adult CNO tendon/ligament/capsular calcification can be seen in costoclavicular or paraspinal ligaments, cartilage of ribs (costochondral or costosternal) and around joints. Severe calcification/ossification may result in complete joint ankylosis. (Figures 3A, 6AB, and 7AB).

### Scintigraphic and CT features of disorders as differential diagnosis for CNO

The main differential diagnoses of CNO are OA, and inflammatory arthritis, which include rheumatoid arthritis (RA), psoriatic arthritis (PsA), and spondylarthritis (SpA). All these disorders are primarily joint disorders associated with joint space narrowing, which is usually asymmetrical in OA, with or without the “vacuum phenomenon” (aseptic gas collection within joints but can also be seen in other adjacent soft tissues due to migration of gas), and usually symmetrical in inflammatory arthritis such as RA. In these arthropathies, polyarticular disease and bilaterality are common and formation of a pannus, as characterized by inflammatory exudate overlying synovial cells on the inside of the joint capsule, is often observed. Secondary adjacent bone involvement may occur, but costoclavicular ligament calcification and ankylosis are not observed. Osteoarthritis is also commonly associated with the presence of osteophytes or bony projections that form along the peripheral margins of joints at the interface between cartilage and the periosteum or in the spine (spondylophytes).

In contrast, CNO primarily affects bone, sparing joint spaces, although these could be eventually secondarily affected due to structural changes and malalignment of bones in adjacent joints. Bone cysts and osteophytes are not primarily observed in CNO, also helping in the differentiation with diseases primarily affecting joints. Isolated involvement of the SCC region is often seen in CNO, and sacroiliac involvement if present is mainly unilateral. Uptake in the mandible is not seen in OA or inflammatory arthritis, and if present may point to a diagnosis of CNO, after exclusion of odontogenic causes.^23-29^

### Statistical analysis

Statistical analysis was performed using IBM SPSS for Windows, Version 25.0 (IBM Corp., Armonk, NY, USA). Results are presented as means (±SD) or as median (range) and categorical variables were summarized using frequency counts and percentages. The inter-observer variability was assessed by using weighted kappa statistics (Cohen’s kappa). Comparison was assessed using unpaired t-tests and chi-square test. Diagnostic odds ratio (OR = exp(beta)) was determined by logistic regression analysis. Sensitivity, specificity, positive predictive value (PPV), and negative predictive value (NPV) were assessed for the identified imaging features. Receiver operating characteristic (ROC) curve and the derived summary measure of accuracy, such as the area under the curve (AUC) as a measurement of a diagnostic performance, were derived by a nonparametric method using IBM SPSS software. Outcomes were corrected for age and level of significance was set at *P* < 0.01 to adjust for multiple testing.

### Ethical approval

The retrieval and analysis of pseudo-anonymized data from patients’ medical records as well as the re-evaluation of CT and skeletal scintigraphy images and analysis of these data were approved by the Medical Ethics Committee of the LUMC. As this was a retrospective cohort study, obtaining individual patients’ informed consent was waived.

## Results

### Demographic and clinical patients’ characteristics

A diagnosis of CNO was confirmed in 118 predominantly female patients (*n* = 103, 87%), with a median age of 36 years (range 17-70) at time of first symptoms, and 45 years (range 20-73) at time of diagnostic imaging, compared to a significantly higher median age of 49 years (range 17-81) at time of first symptoms, and of 53 years (range 17-86) at time of imaging in the 51 non-CNO patients (*P* = 0.002). There was no significant difference in gender (*P* = 0.166) or delay in diagnosis (*P* = 0.792) between CNO and non-CNO patients (Table 1).

**Table 1:** Patient characteristics.

	CNO patients *n* = 118	Non-CNO patients *n* = 51	*P*
Sex			
Male/female	15/103 (13/87%)	11/41 (21/79%)	0.166
Ratio	7:1	4:1	
Age at first symptoms (yrs)	36 (17-70)	49 (17-81)	**0.002**
Age at diagnosis (yrs)	45 (20-73)	53 (17-86)	**0.002**
Delay in diagnosis (yrs)	3.0 (5.3)	2.5 (6.2)	0.792
Main presenting symptoms *n* (%)			
Pain	112 (95%)	47 (92%)	0.345
Inflammatory changes	48 (41%)	30 (59%)	0.045
Bone swelling	36 (31%)	8 (16%)	0.035
Shoulder restriction	42 (36%)	20 (39%)	0.498
Symptoms at diagnosis *n* (%)			
Pain	113 (96%)	47 (92%)	0.098
Inflammatory changes	34 (29%)	26 (51%)	**0.009**
Bone swelling	73 (62%)	13 (25%)	**0.000**
Shoulder restriction	52 (44%)	18 (35%)	0.102
Any autoimmune disease *n* (%)	50 (42%)	11 (22%)	0.502
Pustulosis palmoplantaris	38 (32%)	4 (8%)	**0.002**
Inflammatory markers			
CRP (mg/L) (normal <5.0 mg/L)	7.2 (8.5)	2.8 (6.0)	**0.000**
ESR (mm/h) (normal <20 mm/h)	15.8 (14.1)	11.3 (13.5)	0.026
Bone turnover markers			
ALP (U/L) (normal <98 U/L)	82.0 (23.3)	82.1 (25.5)	0.803
P1NP (ng/mL) (normal <59 ng/mL)	45.1 (19.8)	54.9 (56.8)	0.681
HLA B27 positive	3/33	0/3	0.770

Data expressed as median (range), mean (SD) or number and percentage (%). Significance was set at ***P* < 0.01**.

The main presenting symptom in both groups was pain, predominantly localized in the SCC region in respectively 112 (95%) of CNO and 47 (92%) of non- CNO patients (*P* = 0.345). This was followed by local inflammatory changes in 48 CNO patients (41%), and 30 non-CNO patients (59%) (*P* = 0.05), and restriction of shoulder girdle function in 42 CNO patients (36%) and 20 non-CNO patients (39%) (*P* = 0.498). Thirty-six CNO patients (31%) had a visible bony swelling in the ACW or mandible, compared to 8 non-CNO patients (16%) (*P* = 0.035). In the interval between first presentation and diagnosis, an additional 37 CNO patients (31%) had developed local bone enlargement, compared to a total of 13 non-CNO patients (25%) as documented at diagnosis (*P* = < 0.001), at which time 52 CNO patients (44%) had restriction of shoulder girdle function on the same side as the bony lesion, compared to 18 non-CNO patients (35%) (*P* = 0.102).

Only a limited number of patients (*n* = 33 CNO (29%) and 3 non-CNO patients (6%)), were tested for the HLA-B27 antigen, because of back pain or suspicion of spinal involvement on skeletal scintigraphy. Only 3 CNO patients tested positive (*P* = 0.770), none with radiological evidence for a spondyloarthropathy (Table 1).

### Imaging features

#### Skeletal scintigraphy

Ninety-seven of the 118 CNO patients (82%) demonstrated increased tracer uptake in the SCC region, localized in the majority (*n* = 94, 79.7%) around the SC joint: at the medial end of clavicles, first ribs and manubrium sterni, which was also the main site of increased tracer uptake in non-CNO patients (*n* = 45, 88.2%), *P* = 0.222. Although increased radioactive tracer uptake in the SCC region was predominantly bilateral in both groups, it was less often bilateral in CNO patients (66%) than in non-CNO patients (93.2%), *P* < 0.001. The “classical” bullhead sign was present in only 5 CNO patients (4.2%) and in none of the non-CNO patients. Presence of increased uptake in the clavicle did not significantly differ between groups (25.4% vs 17.4%, *P* = 0.136), but the manubrium sterni was significantly more affected in CNO patients (*n* = 63, 53.4%) compared to non-CNO patients (*n* = 10, 39.2%), *P* < 0.001, OR = 3.79. Increased uptake in the sternal body was present in 19 CNO patients (16.1%), compared to in 4 (7.8%) non-CNO patients (*P* = 0.150). The ribs were affected in both groups, 57.6% in the CNO group vs 51.0% often bilateral, mostly affecting the first rib (*P* = 0.144).

The mandible showed increased uptake in 14 CNO patients (11.9%) compared to 2 non-CNO patients (3.9%) (*P* = 0.062). Patients with increased uptake in the mandible had pain and swelling of the affected site either a dental pathology or changes compatible with CNO on CT scan. Increased tracer uptake in the spine was observed in only 6 CNO patients (5.1%) and in 5 non-CNO patients (9.8%, *P* = 0.875). This was mostly silent although associated with degenerative disk changes on subsequent CT scan (Table 2).

**Table 2:** Prevalence of increased uptake on skeletal scintigraphy.

	CNO patients (*n* = 118)	%	Non-CNO patients (*n* = 51)	%	*P*	OR
SC joint	94	79.7	45	88.2	0.222	0.512
Unilateral	34	36.2	4	8.9	NS	–
Bilateral	**60**	**63.8**	**41**	**91.1**	**<0.001**	**0.064**
Clavicle	30	25.4	9	17.4	0.136	2.043
Unilateral	21	70.0	2	22.2	NS	–
Bilateral	9	30.0	7	77.8	NS	–
Sternal manubrium	**63**	**53.4**	**10**	**39.2**	**0.001**	**3.792**
Sternal body	19	16.1	4	7.8	0.150	2.371
Bullhorn sign	5	4.2	0	0.0	0.998	–
Ribs	68	57.6	26	51.0	0.144	1.715
Unilateral	21	30.9	4	15.4	NS	–
Bilateral	47	69.1	21	80.8	NS	–
Spine	6	5.1	5	9.8	0.875	0.900
Mandible	14	11.9	2	3.9	0.062	4.429
Unilateral	12	85.7	2	100.0	NS	–
Bilateral	2	14.3	0	–	NS	–

Data are expressed as number and percentage; level of significance was set at ***P***** < 0.01**

NS, not significant.

The small number of patients with increased tracer uptake outside the SCC region precludes any reliable conclusion on the ability of scintigraphy to detect silent lesions outside the SCC region.

### CT scans

The manubrium sterni was the most affected site of the ACW in CNO patients: 97 CNO patients (82%) compared to 15 non-CNO (29%) (*P* < 0.001, OR = 9.70). The prevalence of sclerosis and hyperostosis was significantly higher in CNO than non-CNO patients (both features *P* < 0.001).

The region of the sternal body was visualized in only 54% of CNO, and in 49 % of non-CNO patients, with 18 CNO vs 2 non-CNO patients demonstrating sclerosis of the sternal body (*P =* 0.047). Hyperostosis of the sternal body was observed in 12 CNO patients (10%), and in none of the non-CNO patients, *P* < 0.001. Sclerosis and hyperostosis were mainly observed at the location of the first ribs (level 1). There were no significant changes in the second ribs in either group. The clavicle was the second most affected site of the ACW in 95 CNO patients, unilaterally in 40% and bilaterally in 60%, in contrast to non-CNO patients who were predominantly affected unilaterally (73%). The medial end of the clavicle was affected in 81% of CNO patients and 43% of non-CNO patients, respectively (*P* < 0.001). Sclerosis of the clavicle was significantly more prevalent in CNO (81%) compared to non-CNO patients (41%) (*P* < 0.001, OR = 4.11). Hyperostosis of the medial end of clavicles was observed in 62 CNO patients (53%) and in none of the non-CNO patients (*P* < 0.001). Erosive changes were significantly more prevalent in CNO patients, *n* = 36 (31%), than in non-CNO patients, *n* = 5 (10%) (*P* = 0.003, OR = 4.95). The clavicle was also much less affected in non-CNO patients compared to CNO patients, respectively 29 (57%) vs 23 (20%) (*P* < 0.001, OR = 0.23) (Table 3).

**Table 3:** Prevalence of radiological features on CT scanning.

		CNO patients (*n* = 118)	%	Non-CNO patients (*n* = 51)	%	*P*	OR
Clavicle	**Sclerosis**	95	80.5	21	41.2	**<0.001**	**4.110**
	R 1/3	76	64.4	20	39.2	0.030	2.112
	L 1/3	75	63.5	12	23.5	**<0.001**	**3.899**
	Hyperostosis	62	52.5	0	0.0	**<0.001**	NA
	R 1/3	38	32.2	0	0.0	**0.002**	NA
	L 1/3	40	33.9	0	0.0	**0.003**	NA
	Erosions	36	30.5	5	9.8	**0.003**	**4.947**
	None	23	19.5	29	56.9	**<0.001**	**0.232**
Sternal manubrium	Sclerosis	93	78.8	14	27.5	**<0.001**	**9.212**
	Level 1	92	78.0	13	25.5	**<0.001**	**9.699**
	Level 2	20	16.9	3	5.9	NS	NA
	Hyperostosis	80	67.8	0	0.0	**<0.001**	NA
	Level 1	79	66.9	0	0.0	**<0.001**	NA
	Level 2	18	15.3	0	0.0	NS	NA
	Erosions	32	27.1	6	11.8	**0.010**	**3.796**
	Level 1	30	25.4	5	9.8	**0.007**	**4.349**
	Level 2	13	11.0	2	3.9	NS	NA
	None	21	17.8	36	70.6	**<0.001**	**0.087**
Sternal body	Sclerosis	18	15.3	2	3.9	NS	NA
	Level 2	17	14.4	2	3.9	NS	NA
	Hyperostosis	12	10.2	0	0.0	**<0.001**	NA
	None	46	39.0	22	43.1	NS	–
	Not depicted	54	45.8	26	51.0	NS	–
Rib 1	Calcification	8	6.8	4	7.8	NS	NA
	Sclerosis	60	50.8	2	3.9	**<0.001**	NA
	Hyperostosis	75	63.6	0	0.0	**<0.001**	NA
	Hypertrophic ossification	51	43.2	46	90.2	**<0.001**	**0.115**
	C	42	35.6	43	84.3	**<0.001**	**0.116**
	S	45	38.1	42	82.4	**<0.001**	**0.174**
	None	10	8.5	4	7.8	NS	NA
	Not depicted	1	0.8	0	0.0	–	–
Rib 2	Calcification	24	20.3	21	41.2	NS	–
	Sclerosis	7	5.9	0	0.0	NS	NA
	Hyperostosis	11	9.3	0	0.0	NS	NA
	Hypertrophic ossification	5	4.2	2	3.9	NS	NA
	None	46	39.0	8	15.7	**0.004**	**3.550**
	Not depicted	34	28.8	20	39.2	–	–
Spine	Sclerosis	8	6.8	1	2.0	NS	NA
	Hyperostosis	3	2.5	0	0.0	NS	NA
	Erosions	6	5.1	2	3.9	NS	NA
	**Degenerative changes**	**20**	**16.9**	**26**	**51.0**	**<0.001**	**0.245**
Mandible	Sclerosis	5	4.2	0	0.0	NS	NA
	Hyperostosis	4	3.4	0	0.0	NS	NA

Data are expressed as number and percentage; level of significance was set at ***P***** < 0.01**.

NS, not significant; NA, not applicable.

The first ribs were more affected in CNO patients than in non-CNO patients (69 vs 12%, *P* < 0.001, OR = 15.69), bilaterally in 63% of CNO patients and all affected non-CNO patients (*P* < 0.001). Third and fourth ribs were visualized in 47% of CNO patients and 41% of non-CNO patients. Both ribs were very seldom affected in CNO patients, and not affected in non-CNO patients. Sclerosis was also significantly more prevalent in CNO than in non-CNO patients (*P* < 0.001). Hypertrophic ossification was more present in non-CNO patients than in CNO patients, *P* < 0.001, OR = 0.12. Hyperostosis of the ribs was observed only in CNO patients and in none non-CNO patients (*P* < 0.001).

The whole spine was fully visualized with CT images in only 8 CNO patients, 6 of whom had increased radioactive isotope uptake on whole body scintigraphy and 2 were further investigated because of backpain. All 8 patients demonstrated patchy sclerosis of the body of the vertebrae. Degenerative changes of the thoracic spine were also visualized in the absence of increased radioisotope uptake on whole body scintigraphy in 20 CNO and 26 non-CNO patients by being captured by CT scans of the ACW. These changes were significantly more prevalent in non-CNO than CNO patients (*P* < 0.001, OR = 0.25) (Figure 4B).

The mandible was depicted in the 14 CNO patients who had increased uptake in the mandible on skeletal scintigraphy due to a dental pathology or due to CNO in 5 patients who demonstrated unilateral pain and/or swelling of the mandible associated with sclerosis and hyperostosis in four, and only sclerosis in one patient (Figure 2). In the 2 non-CNO patients, who had a CT scan because of unilateral increased tracer uptake on skeletal scintigraphy, no abnormalities were demonstrated on CT scan. The sternoclavicular joint was not affected in 78% of CNO patients, compared to 51% of non-CNO patients (*P* = 0.103).

**Figure 7:**
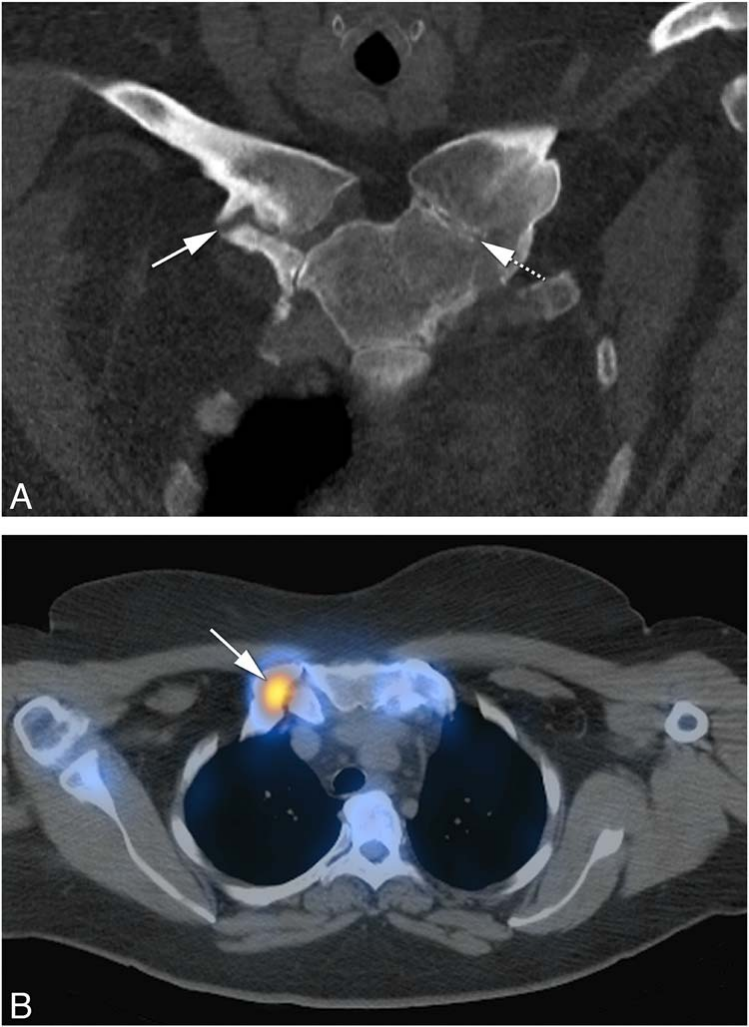
(A) Calcification of the right costoclavicular ligament (arrow) and complete ankylosis of the left costoclavicular joint (dashed arrow). (B) Focal increased uptake at the medial end of the right clavicle and first rib indicating focal active bone formation (arrow). Single photon emission computed tomography of the SCC region showing normal uptake at the left clavicle and sternal manubrium reflecting a more chronic course of the disease at that site.

Calcification/ossification of the SC joints was more prevalent in non-CNO patients (*P* = 0.01) and was mainly unilateral (67%). These calcifications were more likely due to chondrocalcinosis, a common age-related metabolic arthropathy caused by calcium pyrophosphate dihydrate CPPD that can be seen in the SC joint too, especially in the elderly population. Soft tissue swelling of the SC joint was present in both groups, 20 SCCH patients and 15 non-CNO patients (*P* = 0.189, OR = 0.59). Ankylosis of the SC joint was only seen in 4 CNO patients (3%) (Figure 7.), and in none of the non-CNO patients.

Calcification of the costoclavicular ligaments was the most prevalent soft tissue abnormality in CNO patients (*n* = 46, 39%), mainly unilateral in 36 CNO patients, and the prevalence of this feature was significantly higher compared non-CNO patients (*P* < 0.001, OR = 14.61). Ankylosis of the ligaments was only seen in 3 CNO patients (2.5%, all unilaterally).

The manubriosternal joint was visualized in 70% of CNO and in 61% of non-CNO patients, and was affected in 18% and 19%, respectively (not significant). Ankylosis of this joint was observed in 12 CNO and 5 non-CNO patients (*P* = 0.724, OR = 1.23), with clinical swelling on examination in one patient in each group (Table 4 and Figure 3A-C).

**Table 4:** Prevalence of soft tissue changes on CT scanning.

	CNO patients (*n* = 118)	%	Non-CNO patients (*n* = 51)	%	*P*	OR
**SC joint**						
Calcification	4	3.4	10	19.6	**0.010**	**0.191**
Ankylosis	4	3.4	0	0	NS	–
**Costoclavicular ligaments**						
Calcification	46	39.0	2	3.9	**<0.001**	**14.606**
Ankylosis	3	2.5	0	0	NS	–
**Manubriosternal joint**						
Calcification	3	2.5	1	2.0	NS	–
Ankylosis	12	10.2	5	9.8	NS	–
Not depicted	35	29.7	20	39.2	NS	–
**Soft tissue swelling**	20	16.9	15	29.4	NS	–

Data are expressed as number and percentage; level of significance was set at ***P***** < 0.01**.

NS, not significant.

### Relationship between scintigraphic and radiographic features

Computed tomography features of sclerosis, hyperostosis, erosive changes, and/or soft tissue calcification/ossification corresponded to scintigraphic evidence for increased radiopharmacon uptake at affected sites in more than 80% of CNO patients. However, 9.3% of CNO patients (*n* = 11) and 17.6% of non-CNO patients (*n* = 9) had increased tracer uptake without radiological features of hyperostosis, sclerosis, erosive changes or soft tissue ossification, and 18.6% of CNO patients (*n* = 22) and 2.0% of non- CNO patients (*n* = 1) had radiological features of hyperostosis, sclerosis, erosive changes of soft tissue ossification without increased tracer uptake on skeletal scintigraphy.

Chronic non-bacterial osteitis patients were predominantly affected bilaterally (*n* = 78, 66%) on skeletal scintigraphy, and less on CT scan (*n* = 55, 47%). Nearly all non-CNO patients were affected bilaterally on skeletal scintigraphy (*n* = 47, 93%), in contrast to the more unilateral features (*n* = 37, 73%) seen on CT scan.

These discrepancies between scintigraphy and CT scan imaging results, are likely to be due to capturing different stages of the disease at presentation and may represent pitfalls in the diagnosis and decision-making in the treatment of CNO.

### Interobserver variability

The interobserver variability for radiologic and scintigraphic evaluations was substantial among radiologists (kappa = 0.640), poor among nuclear physicians (kappa = 0.131) and fair between radiologists and nuclear physicians (kappa = 0.351). Whereas the discrepancies between nuclear physicians were primarily based on the definition of the bull head sign, the discrepancies between radiologists and nuclear physicians were based on subtle changes of CT scans being generally missed on the low-dose CT of the SPECT-CT investigation, and mild sclerosis, erosive changes or soft tissue calcification being misclassified as degenerative.

After the consensus meetings, there was no difference in interobserver variability among radiologists, and the interobserver agreement among nuclear physicians improved to substantial (kappa = 0.605), and that between radiologists and nuclear physicians to moderate (kappa = 0.464).

### Sensitivity and specificity of imaging tools used in the diagnosis of adult CNO

The sensitivity and specificity of skeletal scintigraphy and CT scan for diagnosing adult CNO were respectively 70% and 72%, and 81% and 94%, at first assessment, and this respectively increased to 79% and 83%, and 87% and 98%, at second consensus assessment. The PPV and NPV for skeletal scintigraphy were 91% and 64%, vs 99% and 80% for CT scan. Combining these two imaging modalities, the sensitivity and specificity increased to respectively 98% and 99% with a PPV of 99% and a NPV of 95% for diagnosing CNO in adults.

The area under the ROC-curve (AUC) showed that increased uptake in the manubrium sterni on skeletal scintigraphy was the only feature that has a predictive ability to discriminate CNO from non- CNO patients (AUC = 0.639, *P* = 0.005). On CT scan, sclerosis of the manubrium sterni had the highest predictive ability to discriminate CNO from non-CNO patients (AUC = 0.757, *P* < 0.001), followed by sclerosis of the first ribs (AUC = 0.735, *P* < 0.001), and clavicles (AUC = 0.655, *P* = 0.001). Hyperostosis of the first ribs has the highest predictive ability to discriminate CNO patients from non-CNO patients (AUC = 0.818, *P* < 0.001), followed by hyperostosis of the manubrium sterni (AUC = 0.810, *P* < 0.001) and clavicles (AUC = 0.729, *P* < 0.001) (Figure S2).

## Discussion

In the present study, we describe the characteristic scintigraphic and radiological features of adult CNO primarily affecting the SCC region and less commonly other regions of the axial skeleton such as mandible, spine, and sacroiliac region in a preselected group of patients from our cohort of adult patients with this rare auto-inflammatory bone disorder.^1^

We identify sclerosis, hyperostosis, bone erosions, and calcification of ligaments and joint capsules leading in some cases to ankylosis of adjacent joints as characteristic CT scan radiological features observed in adult patients with CNO of the SCC region. With sclerosis and hyperostosis of the clavicles, manubrium sterni, and first ribs being distinctive diagnostic features of CNO. In our series, these radiological features were largely associated with concomitant increased uptake of radiopharmacon on skeletal scintigraphy at affected sites, reflecting skeletal inflammatory disease activity. However, it is of note that in the early phases of the disease scintigraphic changes generally precede the development of structural radiological changes (pre-radiographic phase), and that these changes may disappear in the long run as activity of the disease wanes, while radiological features persist (post-scintigraphic phase). In the natural course of the disease, structural radiological and scintigraphic changes coexist in various degrees depending on the stage of the disease. With time, the abnormal structure and increased size of hyperostotic bones may lead to malapposition of adjacent joint surfaces, secondary degenerative changes, and pain and limitation of joint function. The increased size of affected bones may also lead to nerve and vascular compression symptoms.

In patients in whom CNO was excluded, this was based on the finding of reactive sclerosis of the medial end of the clavicles secondary to degenerative changes of the sternoclavicular joint as suggested by the presence of osteophyte and cyst formation and asymmetric joint narrowing.

Erosive changes of the clavicle and upper manubrium were not found to be a specific diagnostic feature of CNO of the SCC region, although they were more prevalent in CNO than non-CNO patients. However, these changes should not be dismissed, as they are likely to reflect inflammatory disease activity, particularly when found in conjunction with other scintigraphic and radiological features of CNO. Hyperostosis of the clavicle, manubrium, and/or first ribs was found to be a characteristic feature of CNO not observed in non-CNO patients.

Hypertrophic ossification is an interesting radiological feature strictly affecting rib cartilage of costochondral and costosternal junctions, mostly of first ribs. The feature is not associated with sclerosis or increased size of bone due to hyperostosis, is not demonstrated to show increased radioisotope uptake, is observed significantly more frequently in our study in non-CNO patients and has the predictive ability to discriminate between CNO and non-CNO patients. This radiological pattern has been previously described as part of the aging process, has been used in the forensic estimation of age, and may represent a pitfall in the diagnosis of CNO especially in the older patient.^19^^,^^20^

Although the spine was not fully visualized by CT scan in the absence of increased tracer uptake on skeletal scintigraphy in most patients, a diagnosis of non-CNO was more likely in the presence of degenerative changes of the thoracic spine as captured by a CT scan of the ACW in non-CNO than in CNO patients.

The radiological features of CNO which were first described as a separate radiographic entity in 1974 by Sonazaki et al.^30^ Since then, one of the main challenges in the diagnosis of CNO remains that clinical symptoms may precede the development of radiological findings, and that radiological features may have already developed before becoming clinically relevant.^6^

Several imaging modalities have been used for the diagnosis of adult CNO. In a systematic review and meta-analysis recently published by our group, the imaging features of bone lesions in the SCC region and the techniques used for diagnosis of CNO were so erratically reported that meta-analytical pooling was not possible.^2^ Based on the results of a recent primary survey conducted among expert stake holders in the management of adult CNO, most of whom were rheumatologists, there was no consensus on the diagnostic imaging tools used in the diagnosis of adult CNO, and no standard diagnostic imaging features used to establish the diagnosis.^31^

Whereas it is widely accepted that CT scan provides greater accuracy in the evaluation of the structural skeletal changes of the ACW in adult CNO, results need to be complemented by whole body scintigraphy or magnetic resonance imaging (MRI) to provide functional imaging of disease activity. Initial imaging solely using whole body MRI has been recently favored, particularly in the evaluation of inflammatory spinal lesions.^32^^,^^33^ Combining results obtained from structural and functional imaging, such as CT, scintigraphy or MRI, with clinical features not only confirms a diagnosis of adult CNO, but also allows the evaluation of disease extent and activity and identifies asymptomatic silent lesions, osteoarticular lesions, and other complications of hyperostosis such as compression symptoms and help decision making about treatment.^5^^,^^7^^,^^34^^,^^35^

In adult CNO, the main purpose of qualitative scintigraphy is not only the detection of active and chronic lesions in the SCC region and other affected sites of the axial skeleton, but also the detection of the “pre-radiographic phase” of the disease, when scintigraphic changes precede the development of structural radiological changes. However, skeletal scintigraphy should not be used as sole tool for diagnosing adult CNO, but should always be used as an adjunct to CT. This tool is very sensitive for early osteoblastic activity, before the development of sclerosis and hyperostosis, and informs about other possible sites of bone lesions, and their activity, thereby reflecting extent and severity of the disease. On the other hand, skeletal scintigraphy is also non-specific, poorly differentiating between adult CNO lesions and other osteo-articular lesions such as OA and inflammatory arthritis due to increased radioisotope uptake around the joints, although in arthritis, increased uptake is seen in and adjacent to joints, whereas in adult CNO increased uptake usually extends beyond the joint space into bones of the axial skeleton: medial end of clavicles, manubrium sterni and sternal body.^36^

It has been argued that the so-called classic “bullhead sign” is both sensitive and specific for adult CNO, and that the specificity of this finding is dependent on the presence of skin lesions, such as pustulosis palmoplantaris.^37^^,^^38^ However, in the present study this was only observed in 4% of our CNO patients. The low prevalence of this sign in our cohort of adult CNO patients is possibly due to our use of more strict criteria for the definition of this sign, or to the fact that patients referred to our Reference Center may have been seen at an earlier stage of their disease. Although specific, this presumed “pathognomonic sign” is in our experience rarely observed in adult CNO and is thus of limited diagnostic value in daily clinical practice.

Surprisingly increased uptake in the sternal manubrium was the only feature on skeletal scintigraphy with a predictive ability to discriminate CNO patients from non- CNO patients (OR = 3.8). Although the prevalence of tracer uptake in the clavicles did not significantly differ between groups, ROC curve and corresponding AUC did indicate a significant predictive ability of uptake at this site for CNO when corrected for age. The discrepancies observed between skeletal scintigraphy and CT scan may be explained by the natural history of the inflammatory disorder. An increased tracer uptake without any associated CT radiological features, as observed in 11 CNO patients, could reflect an early stage of the disease where radiological features without increased tracer uptake, as observed in 22 CNO patients, could possibly reflect a period of remission of the inflammatory process in the presence of residual irreversible radiological changes. These observed discrepancies confirm the need for always acquiring combined information about structural (such as CT or MRI) as well as functional (such as scintigraphy or MRI) changes. Scintigraphy reflects osteoblastic activity, which is the basis of CNO. MRI visualizes signs of active osseous and soft tissue inflammation such as bone marrow edema as well as fat deposition in the bone marrow, a sign of previous inflammation.^5^^,^^7^^,^^34^^,^^35^

In the non-SCCH group, 9 patients demonstrated increased tracer uptake without associated radiological features, which was probably due to degenerative changes with secondary inflammation or due to a primary inflammatory joint disease such as OA. Only 1 non-CNO patients had radiological features without increased tracer uptake due to mild degenerative changes of the SC joint with reactive sclerosis of the adjacent bone.

In practice, if increased uptake is observed on skeletal scintigraphy, the specificity of this tool may be augmented by regional SPECT/CT, which is a full 3D representation of tracer distribution overlaid on a CT for additional information on localized lesions, with respect to anatomical structure and radiological changes.

**Table 5:** Identified scintigraphic and radiological diagnostic features for adult CNO.

	Imaging features for CNO	Features associated with a diagnosis other than CNO
Skeletal scintigraphy	Increased tracer uptake of the sternal manubrium	Bilateral tracer uptake at the SC joints
CT scan	Sclerosis and/or hyperostosis of the sternal manubrium, medial end of clavicles and first ribs Calcification of costoclavicular ligaments	Absence of abnormalities of the clavicles or sternal manubrium Hypertrophic ossification of first ribs Degenerative changes of the spine

Whole body MRI (WB-MRI) is the internationally accepted imaging “gold standard” for the evaluation and monitoring of CNO in children and adolescents because it is radiation-free and has the additional value over CT-scan of being able to detect bone marrow oedema, which reflects active inflammation,^39^^,^^40^ although quantification of inflammatory activity can not be accurately monitored using this imaging tool. WB-MRI does provide information about the distribution of bone lesions in the entire skeleton and detects sites of asymptomatic bone lesions particularly those still confined to the bone marrow.^41^ Recent modifications have been applied by Andreasen et al.^42^ to the Weber score^43^ to include hyperostosis and extension of bone lesions into clavicle and manubrium, and to the Madsen score^44^ to include hyperostosis of the vertebral body.

Although MRI has recently gained favor as the preferred initial imaging modality for suspected CNO also in adults, this tool is more technically challenging than CT scan in studies of the ACW in adults, as breathing movement artifacts may impair the quality of the images, whereas this limitation is less important with the use of WB-MRI in pediatric CNO since avoiding radiation is of paramount importance in this age group, and the involvement of the chest wall is less characteristic of the disease in children and adolescents. MRI is also inferior compared to CT in accurately evaluating some of the characteristic structural morphological changes of CNO such as sclerosis (particularly when subtle), hyperostosis, ligamentous calcifications, erosions and ankylosis in the ACW, which are key to establishing the diagnosis.^23^^,^^45-47^

Notwithstanding these considerations, we believe that the evaluation and monitoring of adult CNO should be based on detailed information regarding structural changes in the ACW as best contributed by CT scans ^42^combined with information regarding disease activity as contributed by MRI or nuclear medicine tools such as whole body scintigraphy or SPECT-CT. More advanced imaging modalities such as [^18^F]NaF-PET/CT may represent significant advantages in the evaluation of adult CNO due to the ability of the technique to combine the high-quality radiological information of CT scans, with the qualitative but also quantitative data on inflammatory bone activity not only for initial assessment but also for monitoring disease activity.

Our study had strengths as well as limitations. Of its main strengths are the inclusion in the study of a relatively large number of well characterized patients with an established diagnosis of adult CNO, from a single expertise center for the disease, with prerequisite availability of complete sets of high quality scintigraphy and CT scans for re-evaluation of images, and with availability of complete sets of clinical data at time of diagnostic imaging including pre-diagnosis historical data, first presenting symptoms and symptoms at diagnosis, and the opportunity to compare adult CNO data with data from a non-CNO reference population also investigated at the same center for suspected adult CNO. A further strength of our study is that all data were systematically and homogeneously collected, using standard in-house developed protocols uniformly used for the acquisition of the consistently high-quality imaging studies performed in our center throughout the years.

A possible limitation of our study is the interobserver variability between investigators for the radiologic and scintigraphic evaluation of imaging scans. However, all evaluations of images were performed by a highly specialized team, with 2 different observers for each modality, blinded for diagnosis and for each other’s evaluation outcomes. The kappa between observers was first only fair but improved after the planned consensus meetings, where specific agreements were reached on specific issues such as the definition of the bullhead sign, and the recognition of “hypertrophic ossification” as a feature not characteristic of SCCH, in contrast to “hyperostosis” which is a prominent feature of the osteitis of CNO/SCCH. The interobserver variability also significantly improved after 2 consensus meetings.

A potential further limitation is that CT scans of mandible and spine were only performed, in patients with increased radiopharmacon uptake at these sites on whole body skeletal scintigraphy.

To the best of our knowledge no validated imaging criteria have been developed to date for the diagnosis of CNO in adult patients, and there are no available studies with a control group of patients presenting with suspected CNO in whom the diagnosis was excluded.

The clinical presentation of adult CNO is heterogenous and different stages of the disease at presentation are associated with a spectrum of clinical and radiological features leading to pitfalls and challenges in diagnosis and management. Identifying and defining unambiguous diagnostic imaging criteria for the disease combining diagnostic structural as well as functional imaging features with a view to improve diagnostic accuracy, has long represented an unmet clinical need that our study has set out to address.

## Conclusion

Our findings identify CT scan features of sclerosis and hyperostosis of manubrium sterni, medial end of clavicles and first ribs, and calcification of costoclavicular ligaments, associated with scintigraphic increased tracer uptake of affected regions, specifically manubrium sterni, as well-defined diagnostic imaging criteria with the best predictive ability for diagnosing adult CNO. We identify discrepancies between scintigraphic and CT scan imaging data, which may represent pitfalls in diagnosis of CNO, and draw attention to the distinctive radiological degenerative feature of “hypertrophic ossification” specifically restricted to (first) ribs as a common pitfall in the diagnosis of adult CNO. We recommend using the identified radiologic diagnostic criteria for the diagnosis of CNO in adults across the spectrum of the disease, from its early to late stages. These identified criteria hold significant clinical implications, not only for establishing early diagnosis of CNO but also for the evaluation of outcome of treatment and its prediction. Future longitudinal studies are required to validate these findings (Table 5).

## Supplementary Material

Supplemental_material-Appendix_A-CNO-SCCH_Radiology_ziae024

Ramautar-Navas_Supplemental_jbmrpl_ziae024

Ramautar-Navas_Supplemental_01_ziae024

Ramautar-Navas_Supplemental_02_ziae024

## Data Availability

The datasets generated and/or analyzed during the current study are available from the corresponding author on reasonable request.
