# Supplementary material for: Defining the imaging diagnostic criteria for adult chronic non-bacterial osteitis
Source: JBMR Plus. 2024 Mar 8;8(5):ziae024. doi: 10.1093/jbmrpl/ziae024 (PMC11008733; doi:10.1093/jbmrpl/ziae024)
Supplement: Supplemental_material-Appendix_A-CNO-SCCH_Radiology_ziae024 [file supplemental_material-appendix_a-cno-scch_radiology_ziae024.docx]

**Supplemental material: Appendix A.** Scoring list for scintigraphic and radiological features

| **Modality** | **Typical radiological findings for SCCH** | | **Present** | | **Notes** | |
| --- | --- | --- | --- | --- | --- | --- |
| **Skeletal scintigraphy *Planar***  **Date:** | ***Increased localized tracer uptake*** *in the region of interest not limited to the joint or without a clear physiological cause (e.g. growth plate):* | | | | | |
|  | SC joint | | R / L / Both | |  | |
|  | Clavicle | | R / L / Both | |  | |
|  | Sternal manubrium | | + / - | |  | |
|  | Sternal body | | + / - | |  | |
|  | Bullhorn sign | | + / - | |  | |
|  | Ribs | | R: 1/2/3/4 L: 1/2/3/4 | |  | |
|  | Spine | | CS / TS / LS | |  | |
|  | Mandible | | R / L / Both | |  | |
|  | Other lesions: | |  | |  | |
| ***SPECT*-CT**  **Date:**  Region:  Extended scan: | SC joint | | R / L / Both | |  | |
|  | Clavicle | | R / L / Both | |  | |
|  | Sternal manubrium | | + / - | |  | |
|  | Sternal body | | + / - | |  | |
|  | Ribs | | R: 1/2/3/4 L: 1/2/3/4 | |  | |
|  | Spine | | CS / TS / LS | | level: | |
|  | Other lesions: | |  | |  | |
|  | **Matching with SCCH** | | **Y/N** | |  | |
| **SPECT-CT or CT**  **Date:**  Region:  Extended scan: | ***Skeletal*** | ***Calcification*** | ***Sclerosis*** | ***Hyperostosis*** | ***Erosive changes*** | ***Hypertrophic ossification*** |
|  | Clavicle | NA | R ⅓ / ⅔ / >  L ⅓ / ⅔ / > | R ⅓ / ⅔ / >  L ⅓ / ⅔ / > | R  L | NA |
|  | Sternal manubrium  Level: | NA | + / -  1 / 2 | + / -  1 / 2 | + / -  1 / 2 | NA |
|  | Sternal body  Level: | NA | + / -  2 / 3 / 4 / 5 | + / -  2 / 3 / 4 / 5 | + / - | NA |
|  | Ribs Right 1 | S / C | S / C | S / C | NA | S / C |
|  | 2 | S / C | S / C | S / C | NA | S / C |
|  | 3 | S / C | S / C | S / C | NA | S / C |
|  | 4 | S / C | S / C | S / C | NA | S / C |
|  | Left 1 | S / C | S / C | S / C | NA | S / C |
|  | 2 | S / C | S / C | S / C | NA | S / C |
|  | 3 | S / C | S / C | S / C | NA | S / C |
|  | 4 | S / C | S / C | S / C | NA | S / C |
|  | Spine (CS / TS / LS) level: |  |  |  |  |  |
|  | Other lesions: |  |  |  |  |  |
|  | ***Soft tissue ossification:*** | ***Normal*** | ***Calcification*** | | ***Ankylosis*** | |
|  | SC joint (intracapsular) | R / L / Both | R / L / Both | | R / L / Both | |
|  | Costoclavicular ligaments | R / L / Both | R / L / Both | | R / L / Both | |
|  | Costosternal joints | R / L / Both | R / L / Both | | R / L / Both | |
|  | Manubriosternal joint | + / - | + / - | | + / - | |
|  | Soft tissue swelling | present Y / N | | | | |
|  | **Matching with SCCH** | **Y / N** | | | | |
| **Other diagnosis** | Arthrosis | Fracture | | | | |
|  | SpA | Congenital disorder | | | | |
|  | Subluxation | Other: | | | | |

**Abbreviations: R** *right;* **L** *left;* **+** *present;* **-** *absent;* **CS** *cervical spine;* **TS** *thoracic spine;* **LS** *lumbar spine;* **S** *sternal;* **C** *costal;* **NA** *not applicable.*
