# Supplementary material for: Defining the imaging diagnostic criteria for adult chronic non-bacterial osteitis
Source: JBMR Plus. 2024 Mar 8;8(5):ziae024. doi: 10.1093/jbmrpl/ziae024 (PMC11008733; doi:10.1093/jbmrpl/ziae024)
Supplement: Ramautar-Navas_Supplemental_jbmrpl_ziae024 [file ramautar-navas_supplemental_jbmrpl_ziae024.docx]

**SUPPLEMENTAL FIGURE LEGENDS**

**Figure S1.** Flowchart of patient inclusion. CNO: chronic non-bacterial osteitis; SCC: sternocostoclavicular; LUMC: Leiden University Medical Center; CT: computed tomography; SPECT-CT: single photon emission computed tomography.

**Figure S2.** ROC curve and corresponding AUC derived from **A:** increased uptake in the manubrium on skeletal scintigraphy as only site with the predictive ability to discriminate CNO from non- CNO subjects (SC joint AUC= 0.451 p=0.316, clavicle AUC=0.572 p=0.140, manubrium AUC=0.639 p0.005, corpus AUC=0.542 p=0.392, bullhead sign AUC=0.522 p=0.660, ribs AUC=0.529 p=0.557, spine AUC=0.476 p=0.622, mandible AUC=0.540 p=0.410), **B:** sclerosis

of the manubrium, first rib and clavicle on CT scanning has the predictive ability to discriminate CNO from non- CNO subjects (clavicle AUC=0,655 p=0.001, manubrium AUC=0.757 p<0.001, corpus AUC=0.557 p0.243, first ribs AUC=0.735 p<0.001), **C:** hyperostosis on CT scanning of the first ribs followed by the manubrium and clavicle has the predictive ability to discriminate CNO from normal subjects (clavicle AUC=0,729 p<0.001, manubrium AUC=0.810 p<0.001, corpus AUC=0.551 p0.295, first ribs AUC=0.818 p<0.001).
