# Supplementary figures and images for: Defining the imaging diagnostic criteria for adult chronic non-bacterial osteitis
Source: JBMR Plus. 2024 Mar 8;8(5):ziae024. doi: 10.1093/jbmrpl/ziae024 (PMC11008733; doi:10.1093/jbmrpl/ziae024)

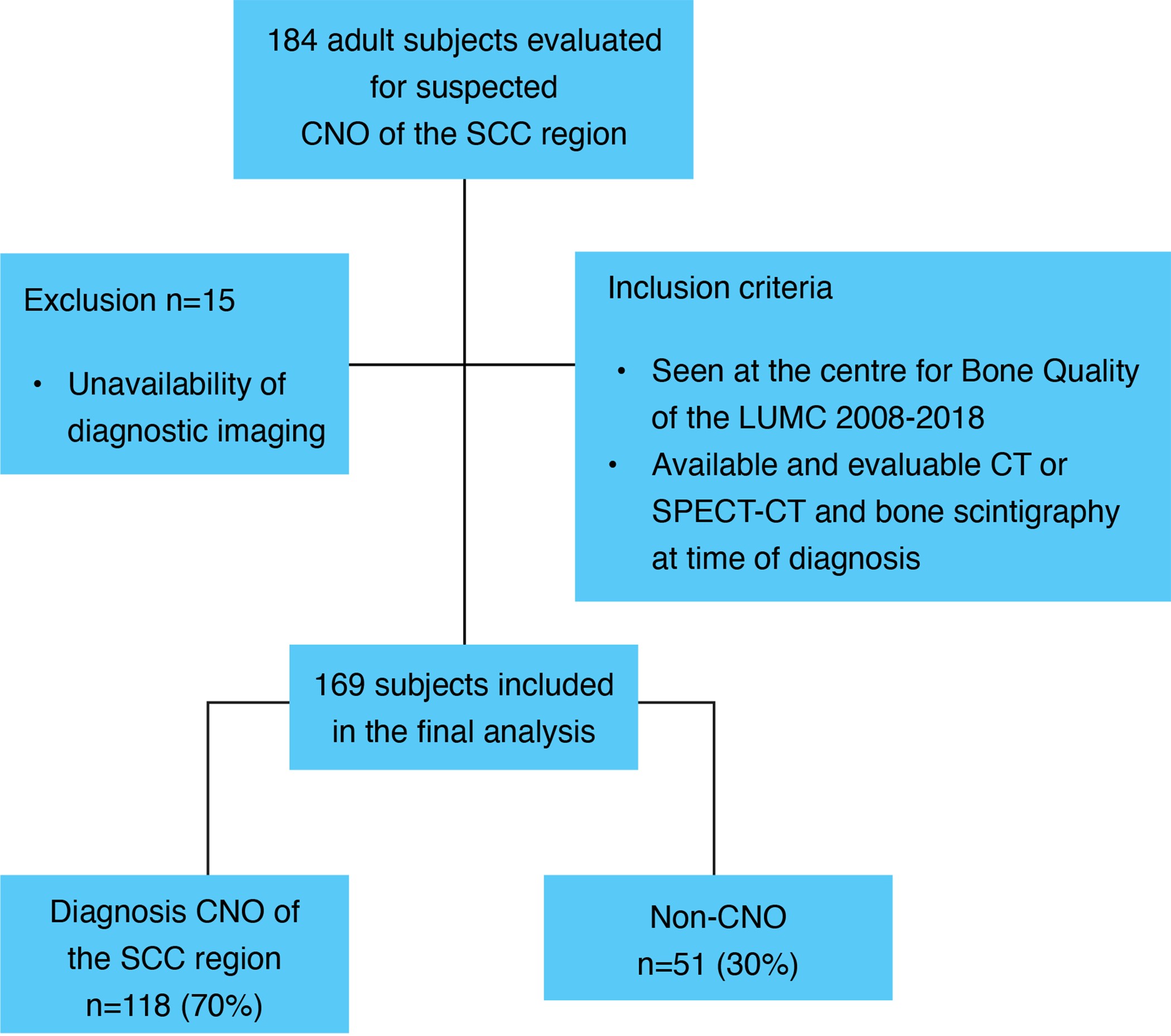

Supplement: Ramautar-Navas_Supplemental_01_ziae024 [file ramautar-navas_supplemental_01_ziae024.jpeg]

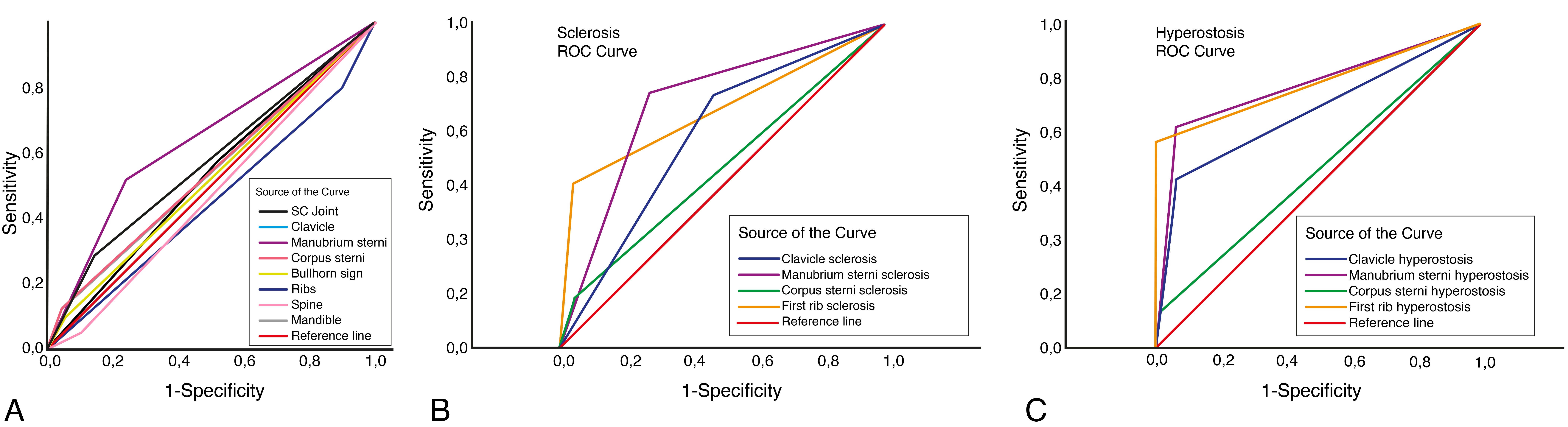

Supplement: Ramautar-Navas_Supplemental_02_ziae024 [file ramautar-navas_supplemental_02_ziae024.jpeg]
